# Mississippi Valley Dental Association

**Published:** 1884-05

**Authors:** F. W. Sage


### MISSISSIPPI VALLEY DENTAL ASSOCIATION.

FORTIETH ANNUAL MEETING, HELD AT CINCINNATI, MARCH 5TH AND 6TH, 1884.

PHONOGRAPHICALLY REPORTED FOR THE INDEPENDENT PRACTITIONER.

BY F. W. SAGE, D. D. S.

(Continued from page 205.)

Upon motion the third and fourth subjects were considered conjointly for discussion. They read as follows: "What progress has been made in arresting and preventing decay of the teeth in the last five years?" "In what respect, if any, are decayed teeth better filled now than ten years ago?"

*Dr. H. A. Smith*—I infer from the wording of the third subject that the committee is of the opinion that we have made no progress during the past five years. I agree that we have made no progress so far as filling teeth with gold is concerned. Fillings were made fifteen or twenty years ago which are to-day absolutely perfect. And what is peculiar, they are apparently as solid as coin. There has been no advancement on that character of operations to this day. In regard to the manner of filling teeth with gold, I do not think there has been much advancement, but there has been a decided advancement made with respect to histological conditions; that is, with respect to protecting the sensitive dentine from the contact of the gold. I think we save many more teeth, by the judicious use of plastics as a foundation for gold, than formerly. I think a man who can make two operations in an hour, which another dentist requires two hours to make, serves his patient just as well, and himself better. There is oftentimes too much excavating done. I have seen students in this college go directly for the pulp, because the tissue was quite soft in that direction. It is not necessary to remove the last vestige of softened dentine. I believe in making simple cavities out of compound ones. Where there is a large portion of gelatinous tissue, I would protect it with a thin film of paraffine. I use, first, an antiseptic application, drying the cavity with the warm-air syringe, wiping out with a little alcohol, drying again, and following this with a little paraffine, which melts at about one hundred and ten degrees Fahrenheit, and when cooled forms a persistent film. We can by that method obviate the necessity, oftentimes, of extensive excavating. Kill the germ of the bacteria, and then fill up the tubules.

[Dr. Smith illustrated on the board the use of plastics, preparatory to the insertion of the gold filling.]

*Dr. G. W. Smith*—My treatment of large cavities is similar to that, excepting that I remove the superincumbent tissues over the pulps that have lost their lime-salts. Then I cap the pulp with oxide of zinc, borax, and creosote.

[To Dr. Hunter's inquiry as to what object he had in using the borax, Dr. Smith returned an answer in explanation, which was apparently vague.]

*Dr. Taft*—One of the signs of progress at the present time is the general diffusion of information, and the general awakening of interest in the matters of hygiene, and the relation existing between the teeth and the health of the system at large. For the last fifty or sixty years these matters have received consideration from men here and there in the profession, but the rank and file have perhaps restricted themselves too much in their investigations to technical matters. I think, too, there has been some progress made in filling teeth of late years. Not only are more new appliances being introduced, but they are being more generally adopted. Formerly there was a more conservative spirit exhibited ; only the bolder ones ventured to adopt new appliances or methods. The increasing numbers of young men entering the profession perhaps accounts for this; then, too, the greater uniformity of the standards by which operations are judged, and the more definite apprehension of just what is required in operations, have led to a quicker appreciation of the suitableness of appliances to the purpose for which they are designed. All this, of course, affects more or less directly the question of preventing decay of the teeth. The treatment of sensitive dentine at one time occupied a large part of the time in our Association. Now, we seldom hear it mentioned. The management of this affection has become comparatively easy. There are so many obtunding agents that we give ourselves no concern about it. Some of the best of these have been introduced within the last two or three years. My attention has been called to *Menthol* for antiseptic purposes. I have been using it also for sensitive dentine. It serves a valuable purpose. I dissolve the crystals to saturation in alcohol and tincture of aconite, and apply to the superficial decay. I have used it for two or three months.

*Dr. Terry*—I have been experimenting for twenty years in the direction of preparing a good obtundent of sensitive dentine, and have been many times disappointed. After long investigation I have succeeded in compounding a medicine, by the use of which an exposed pulp can be saved in nine out of ten cases. By using this preparation upon an exposed pulp, following its use with a capping of iodoform, and this with a cap of oxyphosphate, good results may be expected. It may sometimes be necessary to bleed the pulp slightly. I have used it in my own practice about two years, and have never lost a case.

*Dr. Berry*—For obtunding sensitive dentine I use German creosote, which is superior to the American, and contains no carbolic acid.

*Dr. H. A. Smith*—Dr. Terry's obtundent has been used with satisfactory results in the infirmary of the Ohio Dental College.

Dr. Corydon Palmer was the first to direct attention to the fact that the first annealing of gold foil is the best. Every subsequent annealing seems to impair its value for dental fillings, and to destroy certain desirable qualities. During the past year I have confined myself to the use of unannealed foil, making it relatively cohesive as required. I am satisfied that is the proper way to work with foil. The students in our college have used it in accordance with the idea I have advanced, and I think they almost unanimously confirm Dr. Palmer's theory.

*A Voice*—Are you able to make it sufficiently cohesive for large contours?

*Dr. Smith*—I was for a long time apprehensive that for such cases it could not be as good as cohesive foil. But experience has reassured me. Strips of No. 4 soft foil, annealed and rolled into a rope, may be worked into the cavity without so great liability of the instrument passing through it. It seems to me that I can use it more rapidly, and that less force is required to consolidate it.

*Dr. Van Antwerp*—I have been using unannealed, untrimmed foil, and like it very much better than cohesive foil. It feels soft, like buckskin, and has none of the harsh, crisp qualities of cohesive foil. I have made heavy foil in my rolling-mill by placing gold between sheets of paper and rolling it down. It makes a magnificent heavy foil. I have frequently used aqua regia to etch the surface. Of course the acid must be afterwards removed. I have noticed that a second annealing renders the gold harsh.

*Dr. Jay*—I avoid a second annealing. Use soft foil as such, for filling, in suitable cases. In all cases I want to anneal the foil myself.

*Dr. Taft*—Recommended for large shallow cavities the use of a thin plate of pure gold, made by melting foil scraps and rolling them down to No. 28 or 30, Stub's gauge. Solder on to this a staple, and attach with cement. The question what to use for very soft, frail teeth, is oftentimes a perplexing one. I sometimes say to myself, "what can I do?" I know it will not do to use gold. I have been experimenting with materials to use next to sensitive dentine under gold fillings. My object has been to fill up those imperfectly formed tracts that are shown under a magnifier in many soft teeth. I think varnishing the walls of such cavities is a step in the right direction. Oxy-phosphates, tin, etc., are all very well to use, but after all, we do not want to be restricted to such temporary expedients. We do not enjoy doing these operations over and over, again and again.

*Dr. Callahan*—I have used Robinson's Felt Foil, covering with gold, in approximal cavities. I like the results better than in the cases of many all-gold fillings I have made. This material, where it is not exposed to wear, sometimes discolors. But after a long time I have discovered no sign of softening around the edges of fillings made of it.

*Dr. J. Taft*—I have used tin to a limited extent. My impression is that the Globe Tin Foil of the S. S. White Co. is the best. It has a very peculiar frosted or crystallized surface, and works almost like a plastic. I value it very highly.

*Dr. Wright*—What number do you use?

*Dr. Taft*—About No. 10, I think. It works down like a piece of chamois skin, holds its place well, and takes a smooth finish. I have never used the Robinson filling.

*Dr. Callahan*—There seems to be fear on the part of many who are trying this material that it is not very strong. I have used it for two years, and have had no failures.

*Dr. Van Antwerp*—Can you make gold weld to it?

*Dr. Callahan*—Yes.

*Dr. Emminger*—Stated that in order to make the gold weld to it a layer of the felt foil should be laid upon the portion previously condensed, and upon this the gold should be tacked, and the two pieces worked down together.

*Dr. Taft*—I am glad to hear that, for I am told that some have not succeeded in making gold weld to it.

*Dr. Campbell*—I have succeeded by using No. 30 or 40 gold, but I use sharp-pointed pluggers. Unless it is thoroughly well condensed it is not reliable.

(TO BE CONTINUED.)

